# Clinical and microbiological profile of pneumonia among children with congenital heart diseases at alexandria university children’s hospital

**DOI:** 10.1186/s12879-025-11856-9

**Published:** 2025-12-01

**Authors:** Hani Mahmoud Adel, Marwa Ahmed Meheissen, Asmaa Mahmoud Mohamed Gnina, Nirvana Mahmoud Abdel Fattah, Eman Hamza Hassan

**Affiliations:** 1 Department of Pediatric, Faculty of Medicine, Alexandria University, Alexandria, Egypt; 2 Department of Medical Microbiology & Immunology, Faculty of Medicine, Alexandria University, Alexandria, Egypt

**Keywords:** Congenital heart diseases, Community-acquired pneumonia, RSV, Hospital-acquired pneumonia, Klebsiella.

## Abstract

**Background:**

Pneumonia is a significant cause of morbidity and mortality among children with congenital heart diseases (CHD). Consequently, the current study aimed to identify the prevalent pathogens causing pneumonia among children with CHD, as well as risk factors for mortality among this vulnerable group.

**Method:**

Over a period of one year, all children with CHD who were hospitalized due to pneumonia, including both community-acquired (CAP) and hospital-acquired pneumonia (HAP) were enrolled. Samples of non-bronchoscopic bronchoalveolar lavage (NB-BAL) or induced sputum were collected to identify pathogens using both FTD® (Fast Track Diagnostics) real-time multiplex PCR and traditional culture methods. The VITEK 2 compact system (BioMérieux, Durham, NC, USA) was employed for the complete identification of all bacterial species and for assessing the antimicrobial resistance of all isolated bacteria by culture methods.

**Results:**

The present study included 99 children with CHD and pneumonia; 88.9% had CAP and 11.1% had HAP. Viral pneumonia represented 46.5% of cases; *Respiratory Syncytial Virus* (31.1%), *Rhinovirus* (15.6%), and *adenoviruses* (12.2%) were the most common. Bacterial pneumonia was found in 23.23% of cases; *Klebsiella pneumoniae* (56.3%) and *Streptococcus pneumoniae* (17.2%) were the most common. Out of the studied cases, 75.76% survived and 24.24% deceased. Pulmonary hypertension (95% CI: 1.045–14.907, p=0.027), complications (95% CI: 7.45–162.723, p=0.026), and the need for mechanical ventilation (95% CI: 12.711–207.308, p<0.001) were independent risk factors of mortality.

**Conclusion:**

*RSV*, Klebsiella* pneumoniae* , and *Rhinovirus *are common causes of pneumonia in CHD in our setting. The combination of culture techniques and multiplex PCR was beneficial for the accurate and timely identification of the causative agents of pneumonia in this vulnerable population that helps in guiding antimicrobial therapy.

## Background

Congenital heart disease (CHD) has long been recognized as the most common and severe anomaly at birth [1]. Globally, the prevalence of CHD ranges from 3.7 to 17.5/1000 live births accounting for 30%–45% of all birth defects [2, 3]. The prevalence of CHD differs according to geographical location. In Egypt, the incidence of CHD among Egyptian children has been estimated to be 5–6/1000 live births [4]. Several factors contribute to the higher risk of pneumonia among children with CHD. These include direct impact on the airways, changes in lung mechanics, decreased lung compliance, abnormal physiological mechanisms leading to increased pulmonary blood flow, and congestion that can act as a nidus for lower respiratory tract infections (LRTIs). Additionally, malnutrition with increased metabolic demand and association with other genetic abnormalities can result in impaired immunity, further contributing to severe and recurrent infections [5, 6]. Pneumonia among children with CHD carry high risk of mortality with a rate of 37% compared to 1.5% for those without [7].

In addition to common pathogens causing pneumonia in healthy children, children with CHD are at higher risk of infection with other pathogens such as *Klebsiella pneumoniae*, *Pseudomonas aeruginosa* and *Stenotrophomonas maltophilia* [7]. Identification of pathogens causing pneumonia among children with CHD is a crucial step in the management and improvement of outcome. The current study aimed to investigate the bacterial and viral causes of pneumonia in CHD children. The study also aimed to identify the mortality risk factors in this high-risk group.

## Methods

### Study design and case definition

This prospective study included all pediatric patients with CHD hospitalized due to pneumonia at Alexandria University Children’s Hospital over a period of one year from May 2022 to May 2023. The study included both community-acquired (CAP) and Hospital-acquired pneumonia (HAP). Pneumonia was diagnosed by presence of combinations of any of the following clinical features; fever, cough, tachypnea, difficulty in breathing, increased breathing, hypoxia (Spo2 < 92% in room air), auscultatory findings of pneumonia, accompanied by presences of lung infiltrates in chest radiographs suggestive of pneumonia. Age-specific tachypnea was defined according to the World Health Organization (WHO) guidelines, in children < 2 months respiratory rate ≥ 60 breaths per minute, ≥ 50 breaths per minute in children aged 2–12 months and ≥ 40 breaths per minute in children older than one year [8]. The diagnosis of hospital-acquired pneumonia was established using the pediatric pneumonia criteria from the Centers for Disease Control and Prevention (CDC) and the child showed signs of pneumonia ≥ 48 h following hospital admission, which was not present at the time of admission [9]. Children who underwent surgery for congenital heart diseases were not included in the current study.

The following data was collected from all recruited children: demographic characteristics, vaccination history, presenting symptoms and signs, type of pneumonia and comorbidities (e.g., chromosomal abnormalities). Assessment of nutritional status (Weight, height, body mass index (BMI), and mid-arm circumference) and the World Health Organization z-scores were used to classify the nutritional status of the children [10]. On admission assessment of heart failure and its degree of severity was done based on the modified Ross heart failure classification for children [11]. Chest X-rays: were done for all patients at presentations and were reviewed by a pediatric pulmonologist and a radiologist. Echocardiography was done by a pediatric cardiologist for full assessment of the type and size of the defect, presence of pulmonary hypertension, and the ratio of pulmonary to systemic flow (Qp/Qs ratio) was recorded.

#### Microbiological diagnosis

All samples (Induced sputum or NB-BAL) for microbiological diagnosis were collected before the start of antibiotic treatment and within 24 h of presentation.

### Induced sputum

Before sputum induction, salbutamol inhalation (0.15 mg/kg, maximum dosage 5 mg) was used to prevent bronchospasm followed by saline (3–5%) nebulization with an ultrasonic nebulizer with oxygen flow for 5 min at a time and repeated for 15 min to induce cough. Induced sputum was obtained either by expectoration (in children able to cooperate) or by suctioning through the nasopharynx or oropharynx using the sterile catheter of mucus extractor passed through an appropriate size airway. In mechanically ventilated children, non-bronchoscopic broncho-alveolar lavage (NB-BAL) collected at the time of endotracheal tube insertion. The specimens were collected directly into a sterile mucus extractor container. At least one mL of each sample was aseptically transferred to a falcon tube containing viral transport medium. All samples were transported within 2 h of collection to the Microbiology Laboratory of Alexandria Main University Hospital to be processed [12]. An acceptable sputum sample yielded fewer than 10 squamous epithelial cells and at least 25 polymorphonuclear leukocytes per 100× field. All respiratory samples were cultured onto chocolate, blood, MacConkey’s and Sabouraud dextrose agar plates, further pathogen identification was based on their different morphology and different standard biochemical reactions. Identification to the species as well as antimicrobial susceptibility pattern were performed using Bauer Kirby disc diffusion method and/or VITEK 2 compact automated identification and susceptibility system (BioMérieux, Durham, NC, USA).

The antimicrobial susceptibility pattern was interpreted according to Clinical Laboratory Standards Institute (CLSI 2023) guidelines [13]. Tigecycline disc diffusion interpretation was done according to European Committee on Antimicrobial Susceptibility Testing (EUCAST 2023) [14]. The antimicrobial resistance pattern of all isolated bacteria was further stratified according to Magiorakos et al. into: [15] (1) Multidrug-resistant (MDR): organisms that acquire non-susceptibility to at least one agent in three antimicrobial categories. (2) Extensively drug-resistant (XDR): organisms that are not susceptible to at least one agent in all but two or fewer antimicrobial categories. (3) Pan drug-resistant (PDR): organisms that are non-susceptible to all agents in all antimicrobial categories.

### Real time multiplex PCR for detection of pneumonia pathogens

An equal volume of N-acetyl-L-cysteine (10 g/L) was added to sputum samples and shaken at room temperature for 30 min to be fully liquified. Nucleic acid extraction from liquified Sputum and NB-BAL samples using the universal extraction kit (GeneAII EXGENE, Korea) according to the manufacturer’s instructions. The real time PCR amplification and detection of nucleic acid was performed by Real time multiplex PCR [16] using the FTD^®^ (Fast Track Diagnostics). Respiratory Pathogens 33 assay for the simultaneous amplification of 12 bacterial pathogens, including: *Mycoplasma pneumoniae*,* Chlamydia pneumoniae*,* Streptococcus pneumoniae*, *Hemophilus influenzae type B*, *Hemophilus influenzae (non-B)*,* Staphylococcus aureus*, *Moraxella catarrhalis*,* Bordetella pertussis*,* Bordetella parapertussis*,* Klebsiella pneumoniae*, *Legionella pneumophila*,* and Salmonella species* as well as of 19 viruses, including: *influenza A virus*,* influenza B virus*,* influenza C virus*,* influenza A(H1N1) virus (swine-lineage)*,* human parainfluenza viruses 1*,* 2*,* 3 and 4*,* human coronaviruses NL63*,* 229E*,* OC43 and HKU1*, *Human metapneumoviruses A/B*, *Human Rhinovirus*,* human Respiratory Syncytial Viruses A/B*,* Human Adenovirus*,* Enterovirus*,* Human Parechovirus*,* Human Bocaviru*s. The results were considered positive if cycle thresholds (Ct) are ≤ 40.

Detection of organisms by PCR is considered infection rather than colonization based on existence of pneumonia symptoms and radiological evidence of pneumonia on chest X ray (pulmonary infiltrates). After identifying the causative agents, pneumonia was further classified as viral pneumonia, bacterial pneumonia or viral bacterial co-infection if both viral and bacterial pathogens were detected.

### Statistical analysis

Quantitative data were described by mean (standard deviation) or Median (Interquartile range) as appropriate. While categorical variables were summarized by frequency and percent. Bivariate analysis using Mann-Whitney test based for quantitative variables distribution as well as Pearson’s Chi-square test compared different demographic and clinical parameters between survivors and non-survivors. Fisher exact (FEp) and Monte-Carlo significance (MCp) were performed if more than 20% of total expected cell counts < 5. Statistically significant, and clinically relevant covariates were fitted in multivariate stepwise backward Wald logistic regression analysis. Statistical analysis was done using IBM SPSS statistics program version 29. All statistical tests were two-sided and judged at 0.05 significance level.

## Results

**Fig. 1:**
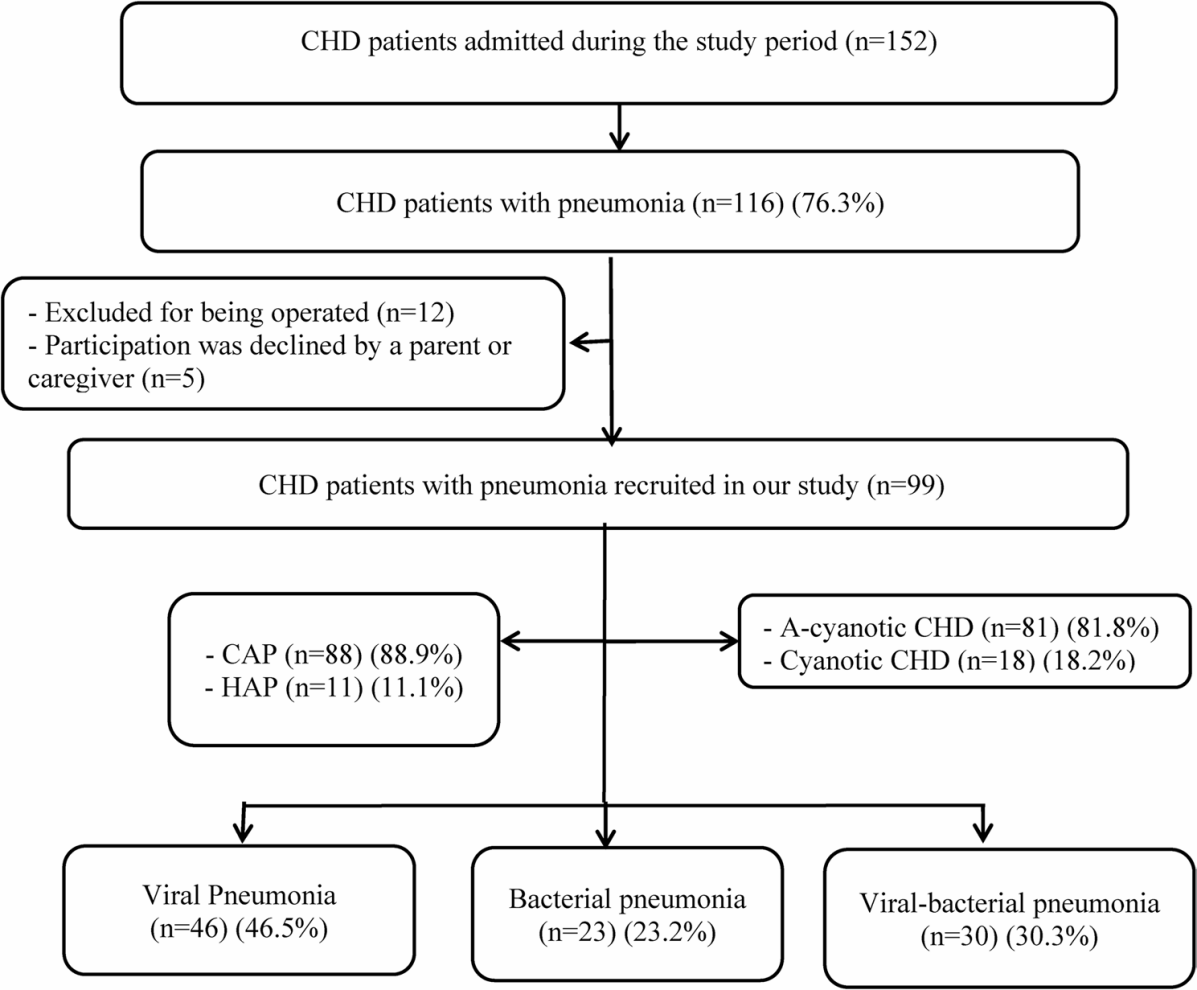
RECORD diagram of the population studied.CHD: Congenital heart diseases, CAP: Community-acquired pneumonia, HAP: Hospital-acquired pneumonia

During the study period, 152 children with CHD were admitted to hospital; 99 of these patients were found to be eligible for inclusion. Of the cases studied, 88.9% (*n* = 88) had community-acquired pneumonia, while 11% (*n* = 11) had hospital-acquired pneumonia. All HAP were late onset (symptoms developed 5 days following admission), and every child had one episode (Fig. 1). Males were predominant, accounting for 58.6% (*n* = 58). Infants under one year of age constituted the largest age group, accounting for 79.8% (*n* = 79). One third (30.3%) of children were exclusively breastfed, and 36.4% (*n* = 36) were malnourished. Only 5% (*n* = 5) of cases were unvaccinated and the rest of studied children were vaccinated up to date according to the compulsory vaccine scheduled in Egypt that include only *Haemophilus influenzae* vaccination. 33.3% (*n* = 33) had underlying comorbidities with down syndrome was the most prevalent presented in 22.2% (*n* = 22) of children (Table 1).

**Table 1:** Clinical characteristics and outcome of children with CHD admitted with pneumonia

	**Total (** ***n*** ** = 99)**	**Cause of pneumonia**			***p*** value
		**Viral (** ***n*** ** = 46) (46.5%) **	**Bacterial (** ***n*** ** = 23) (23.2%) **	**Viral-bacterial coinfection (** ***n*** ** = 30) (30.3%) **	
**Sex**					
Male	58 (58.6%)	25 (54.3%)	14 (60.9%)	19 (63.3%)	0.716
Female	41 (41.4%)	21(45.7%)	9 (39.1%)	11 (36.7%)	
**Age (months)**					
**Median (IQR)**	4.0(2.0-9.0)	4.0(2.0-9.0)	4.0 (2.0-10.5)	3.25 (2.0-8.0)	0.640
<12	79 (79.8%)	36 (78.3%)	18 (78.3%)	25 (83.3%)	0.954
12 – <60	19 (19.2%)	9 (19.6%)	5 (21.7%)	5 (16.7%)	
≥60	1(1.0%)	1 (2.2%)	0 (0.0%)	0 (0.0%)	
**Breastfeeding**	30 (30.3%)	9^a^ (19.6%)	10^b^ (43.5%)	11^ab^ (36.7%)	0.083
**Preterm**	13 (13.1%)	7 (15.2%)	2 (8.7%)	4 (13.3%)	0.866
**Residence**					
Urban	58 (58.6%)	34^a^ (73.9%)	11^b^ (47.8%)	13^b^ (43.3%)	0.015
Rural	41 (41.4%)	12^a^ (26.1%)	12^b^ (52.2%)	17^b^ (56.7%)	
**Malnutrition**	36 (36.4%)	10 (21.7%)	11 (47.8%)	15 (50.0%)	0.019
Unvaccinated*	5 (5.1%)	0 (0.0%)	5 (21.7%)	0 (0.0%)	<0.001
**Co-morbidities**	33 (33.3%)	10 (21.7%)	12 (52.2%)	11 (36.7%)	0.037
**Down syndrome**	22(22.2%)	10 (21.7%)	5 (21.7%)	7 (23.3%)	0.985
**Type of CHD**					
A-cyanotic	81 (81.8%)	38 (82,6%)	20 (87.0%)	23 (76.7%)	0.618
Cyanotic	18(18.2%)	8 (17.4%)	3 (13.0%)	7 (23.3%)	
**Type of pneumonia**					
CAP	88(88.9%)	46 (100.0%)	16 (69.6%)	26 (86.7%)	<0.001
HAP	11 (11.1%)	0 (0.0%)	7 (30.4%)	4 (13.3%)	
**Fever**	88 (88.9%)	37(80.4%)	22 (95.7%)	29 (96.7%)	0.063
**Cough**	61(61.6%)	41 (89.1%)	9 (39.1%)	11 (36.7%)	0.001
**Tachypnea**	86(86.9%)	34 (73.9%)	23 (100.0%)	29 (96.7%)	0.002
**Cyanosis**	37 (37.4%)	10(21.7%)	11 (47.8%)	16 (53.3%)	0.010
**Apnea **	5 (5.1%)	0 (0.0%)	2 (8.7%)	3 (10.0%)	0.046
**Wheezes**	41(41.4%)	22 (74.8%)	4 (17.4%)	15 (50.0%)	0.028
**Crackles **	77 (77.8%)	37(80.4%)	17 (73.9%)	23 (76.7%)	0.815
**Extra-pulmonary symptoms**	35(35.4%)	14 (30.4%)	11 (47.8%)	10 (33.3%)	0.349
**Heart failure**	30 (30.3%)	8(17.4%)	10 (43.5%)	12 (40.0%)	0.032
**Need for oxygen supplementation**	69 (69.7%)	28(60.9%)	18 (78.3%)	23 (76.7%)	0.203
**Need for mechanical ventilation (MV)**	30 (30.1%)	6 (13.0%)	14 (60.9%)	10 (33.3%)	<0.001
**Duration of MV (Median) (IQR)**	7.0 (5.0-11.0)	6.5(6.0-8.0)	6.50(4.0-14.0)	8.50(6.0-10.0)	0.629
**Hospital stay (Median) (IQR)**	8.0(7.0-12.0)	7.0 (6.0-9.0)	10.0(7.0-14.5)	10.0(7.0-14.0)	0.003
**Fate**					
Improved	75(75.8%)	43 (93.5%)	11 (47.8%)	21 (70.0%)	<0.001
Died	24 (24.2%)	3 (6.5%)	12 (52.2%)	9 (30.0%)	

*IQR *Interquartile range, *CHD *Congenital heart diseases, *CAP *Community -acquired pneumonia, *HAP *Hospital-acquired pneumonia, 11 cases of Viral pneumonia had more than one virus isolated, Bacterial pneumonia: 3 cases had combined bacterial coinfection, * in Egypt only Haemophilus influenzae vaccine included in routine immunization and pneumococcal vaccine not part of the vaccination schedule

Statistically significant at p value ≤ 0.05

The majority (81.8%) of studied cases had a-cyanotic heart diseases with ventricular septal defect (VSD) (44.4%), atrial septal defect (ASD) (19.8%) and atrioventricular septal defect (AVSD) (18.5%) were the most common. Furthermore 18.2% (*n* = 18) had cyanotic CHD with tetralogy of Fallot (61.1%) is the most common (Table 2).

**Table 2:** Distribution of studied cases according to common types of congenital heart diseases

**Type of CHD**	**(n) (%) **
**A cyanotic CHD **	**81 (81.8%) **
Ventricular septal defect (VSD)	36 (44.4%)
Atrial septal defect (ASD)	16 (19.8%)
Atrioventricular septal defect (AVSD)	15 (18.5%)
Patent ductus arteriosus (PDA)	3 (3.7%)
VSD & ASD	3 (3.7%)
VSD & PDA	2 (2.5%)
VSD & ASD & PDA	1 (1.2%)
AVSD & pulmonary stenosis (PS)	3 (3.7%)
VSD & PS	1 (1.2%)
ASD & PS	1 (1.2%)
**Cyanotic CHD**	**18 (18.1%)**
Tetralogy of Fallot (TOF)	11 (61.1%)
Double outlet right ventricle (DORV)	3 (16.7%)
Transposition of the great arteries (TGA)	3 (16.7%)
Total Anomalous Pulmonary Venous Return (TAPVR)	1 (5.6%)

Pneumonia was classified based on the causative agent into viral pneumonia (46.5%), viral-bacterial pneumonia (30.3%), and bacterial pneumonia (23.2%). Most **(**73.9%) children with viral pneumonia lived in urban areas, whereas 52.2% of those with bacterial pneumonia were from rural regions which was statistically significant (*p* = 0.015). In terms of clinical presentation, Fever (88.9%), tachypnea (86.9%), cough (61.6%), and wheezing (41.4%) were the most common. The presenting symptoms of pneumonia exhibited a significant difference between viral and bacterial pneumonia; tachypnea was seen in all cases of bacterial pneumonia and in 73.9% of viral pneumonia (*p* = 0.002). Children with viral pneumonia experienced coughing more frequently (89.1%) than those with bacterial pneumonia (39.1%) (*p* < 0.001). Furthermore, viral pneumonia had a significantly higher prevalence of wheezing (74.8%; p0.028). Nearly one third (30.3%) of children are presented with heart failure at the onset of pneumonia. Children with bacterial pneumonia were significantly (*p* < 0.001) more likely to require ventilator support (60.9%). They also had significantly longer duration of hospital stay [10.0(7.0–14.5.0.5) days; *p* = 0.003] (Table 1).

**Fig. 2:**
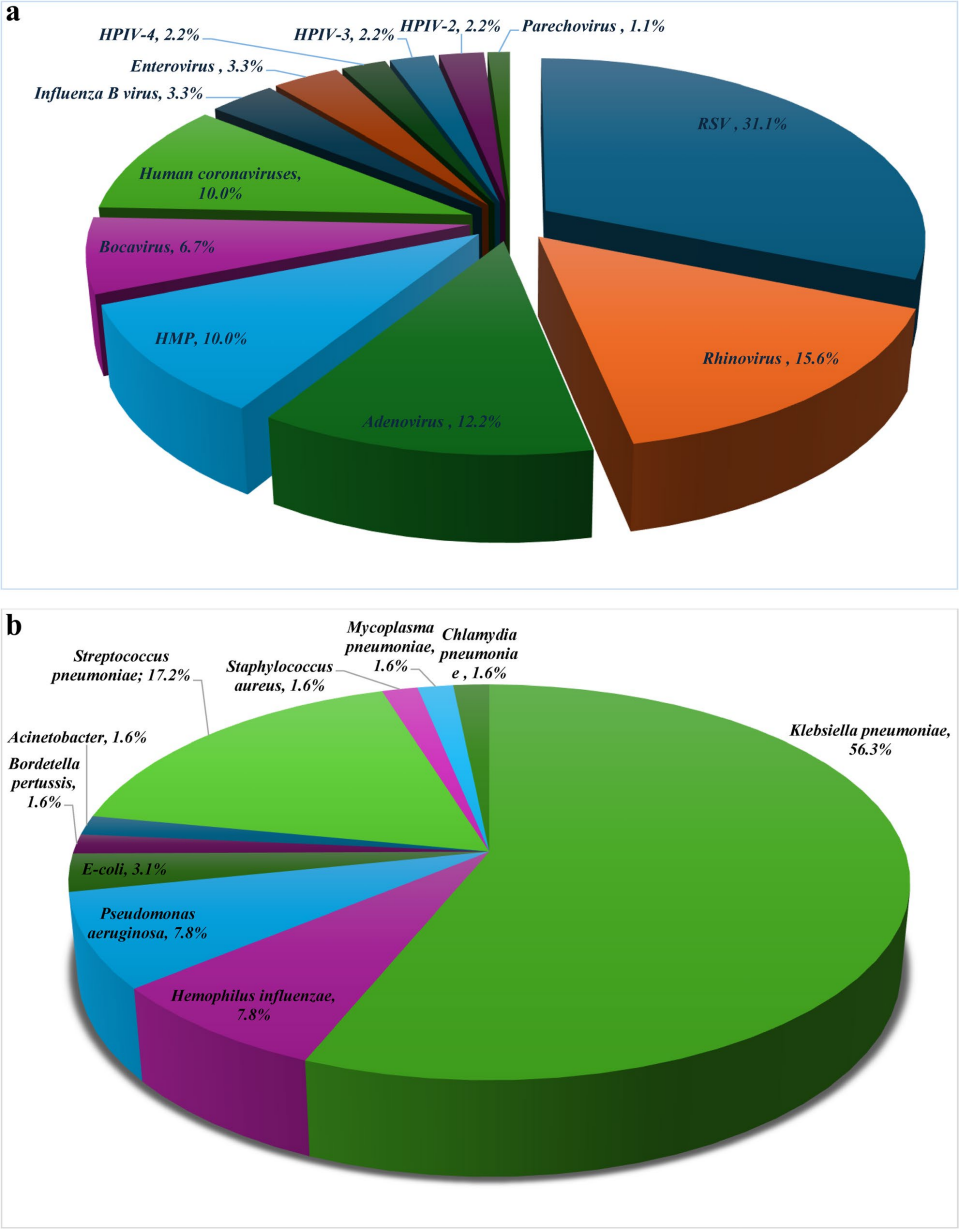
**a** Common viral pathogens causing pneumonia among children with CHD (*n*=90). Respiratory Syncytial Virus (RSV, *n*=28), Rhinovirus (*n*=14), Adenovirus (*n*=11), Human Metapneumovirus (HMPV, *n*=9), Bocavirus (n-6), Human Coronaviruses (*n*=9) [229 (*n*=3), HKU1 (*n*=5), NL63 (*n*=1)], Influenza B (*n*=3), Enterovirus (*n*=3), Human Parainfluenza-4 Virus (HPIV-4, *n*=2), Human Parainfluenza-3 Virus (HPIV-3, *n*=2), Human Parainfluenza-2 Virus (HPIV-2, *n*=2), Parechovirus (*n*=1). **b** Common bacterial pathogens causing pneumonia among children with CHD (n=64). Klebsiella pneumoniae (n=36), Streptococcus pneumoniae (n=11), Pseudomonas aeruginosa (n=5), Hemophilus influenzae (n=5), E. coli (n=2), Acinetobacter baumannii, Methicillin Resistant Staphylococcus aureus, Bordetella pertussis, Mycoplasma pneumoniae (n=1), Chlamydia pneumoniae (n=1 )

The common causative agents of pneumonia are demonstrated in Fig. 2a and b. Out of 99 pneumonia cases, 46 (46.5%) viral pneumonia [35 cases mono-viral, 9 cases bi-viral, 2 cases triple virus infection], 23 (23.2%) bacterial pneumonia [20 cases mono-bacterial, 3 cases poly-bacterial infection] and 30 (30.3%) viral-bacterial co-infection were identified (Fig. 1).

A total of 90 viruses and 64 bacteria were isolated from the 99 pneumonia cases. Out of 90 viruses identified, *RSV* (28/90; 31.1%), *Rhinovirus* (14/90; 15.6%), and *Adenovirus* (11/90; 12.2%) were the most prevalent. All viral pneumonia were community acquired (Fig. 2a).

Out of 64 isolated bacteria, Gram-negative bacteria constituted 78.1% (50/64), while gram-positive bacteria 18.8% (12/64 isolates), and atypical bacteria represented only 3.1% (two isolates) (Fig. 2b; Table 3). Multiplex PCR improved the identification of bacteria causing pneumonia, identifying 87.5% (*n* = 56) of bacterial isolates. 54.5% of *Streptococcus pneumoniae* were detected by bacterial culture while all isolates were *identified* by PCR. PCR was the only diagnostic technique for *Chlamydia pneumoniae*,* Mycoplasma pneumoniae*,* Hemophilus influenzae* and *Bordetella pertussis* as their culture methods were not included in the current study (Table 3). Among cases of bacterial CAP (*n* = 50), *Klebsiella pneumoniae* (26/50; 52%), *Streptococcus pneumoniae* (11/50; 22%) and *Haemophilus influenzae* (5/50; 10%) were the most common. While *Klebsiella pneumoniae* (10/14; 71.4%), *Pseudomonas aeruginosa* (3/14; 21.4%) and *Acinetobacter baumannii* (1/14; 7.1%) were the primary bacterial causes of HAP (*n* = 14). Out of 41 bacteria isolated by culture, 24 (21 CAP, 3 HAP) were MDR and 9 (8 HAP, one CAP) were XDR. Regarding CAP, MDR bacteria were the most common accounting for 72.4%, while XDR represented 66.7% of HAP isolates (*p* = 0.001) (Table 4).

**Table 3:** Distribution of isolated bacterial pathogens causing pneumonia by culture and PCR in patients with CHD (*n*=64)

**Bacterial pathogens**(***n***=64)	**Positive culture **	**Positive PCR **	**Positive culture and PCR **
	**No (%)**	**No (%)**	**No (%)**
**Gram-negative bacteria (** ***n*** **=50)**			
* Klebsiella pneumoniae *(*n*=36)	26 (72.2%)	36 (100%)	26 (72.2%)
* Pseudomonas aeruginosa* (*n*=5)	5 (100%)	0	0
* Hemophilus influenzae *(*n*=5)	0	5 (100%)	0 (0%)
* E-coli *(n=2)	2 (100%)	0	0
* Acinetobacterbaumannii* (*n*=1)	1 (100%)	0	0
* Bordetella pertussis *(*n*=1)	0	1 (100%)	0
**Gram-positive bacteria (12)**			
* Streptococcus pneumoniae *(***n***=11)	6 (54.5%)	11 (100%)	6 (54.5%)
* Methicillin resistant Staphylococcus aureus *(*n*=1)	1 (100%)	1(100%)	1 (100%)
**Atypical bacteria (** ***n*** **=2)**			
* Mycoplasma pneumoniae *(*n*=1)	0	1 (100%)	0
* Chlamydophila pneumoniae *(*n*=1)	0	1 (100%)	0
**Total**	41 (64.1%)	56 (87.5%)	33 (51.6%)

*PCR* Polymerase chain reaction

**Table 4:** Comparison between community-acquired and healthcare-associated pneumonia in patients with CHD

	**Type of pneumonia**		***p *** **value**
	**CAP ** **(n = 88) (88.9%)**	**HAP ** **(n = 11) (11.1%)**	
**Need for mechanical ventilation **	21 (23.9%)	9 (81.8%)	<0.001
**Complications**	33 (37.5%)	10 (90.9%)	0.001
**Heart failure**	24 (27.3%)	6 (54.5%)	0.084
**Fate**			
** Improved**	73 (83.0%)	2 (18.2%)	<0.001
** Died**	15 (17.0%)	9 (81.8%)	
**Median days of hospital stay **	8.0 (1.0 – 45.0)	12.0 (4.0 – 31.0)	0.078
**Bacterial pneumonia (n=64)**	**50 (79.2%)**	**14 (20.8%)**	
**Gram-negative bacteria (n=50) **	36 (72.0%)	14 (100%)	0.025
*Klebsiella pneumoniae* (n=36)	26 (52.0%)	10 (71.4%)	0.193
*Haemophilus influenzae *(n=5)	5 (10.0%)	0	0.218
*Pseudomonas aeruginosa *(n=5)	2 (4.0%)	3 (21.4%)	0.031
*E-coli *(n=2)	2 (4%)	0	0.447
*Bordetella pertussis *(n=1)	1 (2.0%)	0	0.596
*Acinetobacter baumannii *(n=1)	0	1 (7.1%)	0.057
**Gram- positive bacteria (=12) **	12 (18.75%)	0	0.042
*Streptococcus pneumoniae (n=11)*	11 (22.0%)	0	0.053
*Methicillin resistant Staphylococcus aureus (n=1)*	1 (2.0%)	0	0.596
**Atypical bacteria (n=2)**	2 (3.1%)	0	0.447
*Mycoplasma pneumoniae (n=1)*	1 (2.0%)	0	0.596
*Chlamydia pneumoniae (n=1)*	1 (2.0%)	0	0.596
**Antibiotic resistance pattern of culture positive cases (n=41)**			0.001
Susceptible (*n*=8)	7 (24.1%)	1 (8.3%)	
Multi-drug resistant (MDR) (n=24)	21 (72.4%)	3 (25%)	
Extended-drug resistant (XDR) (n=9)	1 (3.4%)	8 (66.7%)	

*CAP *Community-acquired pneumonia, *HAP *Hospital-acquired pneumonia, statistically significant at *p* value ≤ 0.05

**Fig. 3:**
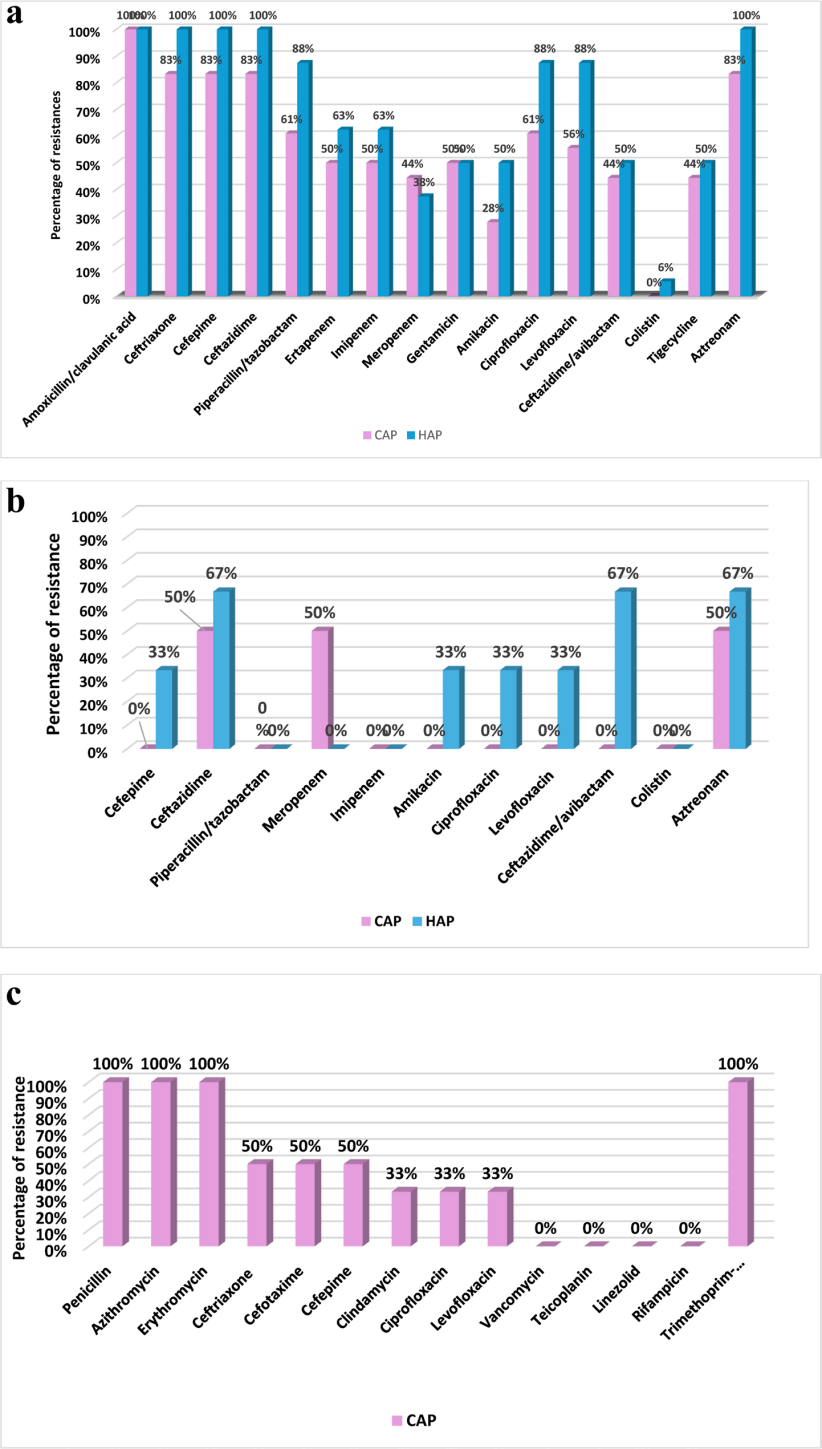
**a** Antimicrobial resistance pattern of *Klebsiella pneumoniae. ***b** Antimicrobial resistance pattern of *Pseudomonas aeruginosa. ***c** Antimicrobial resistance pattern of *Streptococcus pneumoniae*

*Klebsiella pneumoniae* exhibited high rates of antimicrobial resistance. *Klebsiella pneumoniae* in CAP showed the lower resistance to amikacin (28%), meropenem (44%), ceftazidime-avibactam (44%), and tigecycline (44%). While, imipenem, piperacillin/tazobactam, and 3rd and 4th generation cephalosporins showed the highest resistance rates (50–83%). *Klebsiella pneumoniae* in HAP cases showed resistance rates ranging from 38 to 50% to meropenem, aminoglycosides, ceftazidime-avibactam and tigecycline. Although, Colistin showed the least resistance rate (6%), this was alarming (Fig. 3a).

**Fig. 4:**
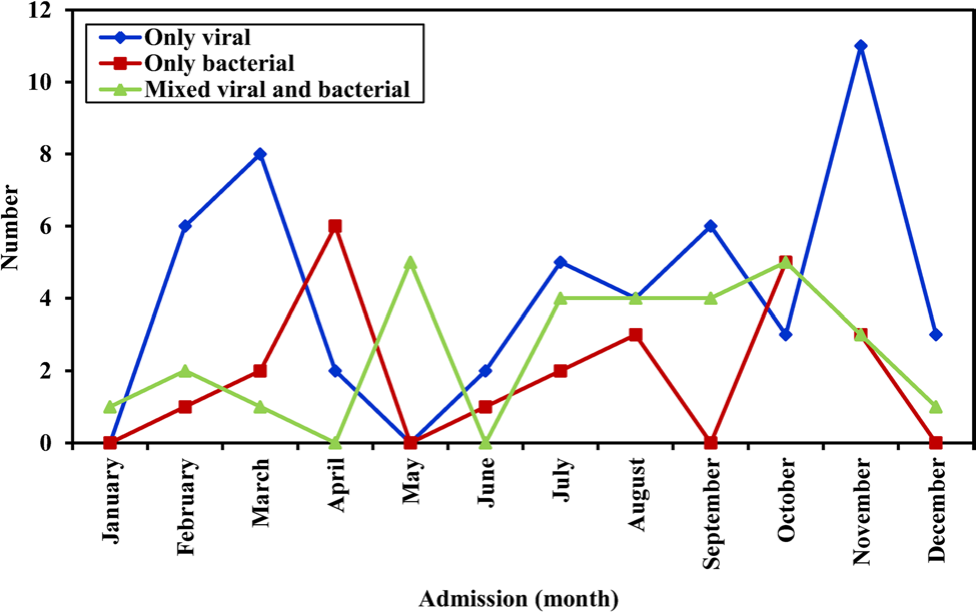
Seasonal variation of the three types of pneumonia

*Pseudomonas aeruginosa* CAP was susceptible to most of tested antibiotics but 50% of isolates were resistant to meropenem, ceftazidime and aztreonam. In contrast, HAP isolates showed greater resistance than CAP: 67% for ceftazidime, ceftazidime-avibactam, and aztreonam. 33.3% of isolates exhibited resistance to aminoglycosides and quinolones; nonetheless, all HAP isolates were sensitive to meropenem, piperacillin/tazobactam and colistin (Fig. 3b). All isolates of *Streptococcus pneumoniae* were community-acquired and were susceptible to vancomycin, linezolid, and rifampicin, while 33.3% were resistant to each of clindamycin, ciprofloxacin, and levofloxacin, 50% of isolates were resistant to third-generation cephalosporins, and all isolates were resistant to macrolides and penicillin (Fig. 3c). Viral pneumonia demonstrated two peaks in February-March and November. viral-bacterial co-infection showed peak incidence in May and October (Fig. 4).

Concerning the outcome, 75.8% (*n* = 75) of the studied patients survived, while 24.2% (*n* = 24) were deceased. Comparing between the two groups; co-morbidity, malnutrition, and presence of pulmonary hypertension were significantly higher among deceased children (*P* = 0.047, *P* < 0.001, and *p* = 0.033 respectively). In terms of clinical presentation, all deceased children experienced tachypnea and difficulty in breathing upon admission, and this was statistically significant (*P* = 0.025 and 0.019 respectively). Additionally, 70.8% and 16.7% of deceased children also had cyanosis and apnea respectively on admission and these findings were statistically significant (*P* < 0.001, 0.012, respectively) (Table 5).

**Table 5:** Comparison between survived and deceased children with CHD and pneumonia

		**Fate**		***p*** ** value**
		**Survived (n=75) (75.8%)**	**Deceased (n=24) (24.2%)**	
**Age (months) **	**<12 months**	59 (78.7%)	20 (83.3%)	0.830
	**12-60 months**	15 (20.0%)	4 (16.7%)	
	**>=60 months**	1 (1.3%)	0 (0.0%)	
**Sex**	**Males**	46 (61.3%)	12 (50.0%)	0.327
	**Females**	29 (38.7%)	12 (50.0%)	
**Type of CHD**	**A cyanotic**	64 (85.3%)	19 (79.2%)	0.475
	**Cyanotic**	11 (14.7%)	5 (20.8%)	
**Co-morbidity **	Yes	21 (28.0%)	12 (50.0%)	0.047
**Malnutrition**	Yes	56 (74.7%)	7 (29.2%)	<0.001
**Pulmonary hypertension**	Yes	30 (44.1%)	3 (16.7%)	0.033
**Fever **	Yes	67 (89.3%)	21 (87.5%)	0.725
**Cough **	Yes	54 (72.0%)	7 (29.2%)	0.001
**Tachypnea **	Yes	54 (72.0%)	24 (100%)	0.025
**Difficulty in breathing **	Yes	62 (82.7%)	24 (100%)	0.019
**Cyanosis **	Yes	20 (26.7%)	17 (70.8%)	<0.001
**Apnea **	Yes	1 (1.3%)	4 (16.7%)	0.012
**Wheezes **	Yes	35 (46.7%)	6 (25.0%)	0.061
**Crackles **	Yes	54 (72.0%)	23 (95.8%)	0.015
**Complications **	Yes	18 (24.0%)	22 (91.7%)	<0.001
**Heart failure **	Yes	14 (18.7%)	16 (66.7%)	<0.001
**WBC x 10** ^9^ **/L**	**Leukocytosis**	11 (14.7%)	6 (25.0%)	0.344
	**Leucopenia**	3 (4.0%)	0 (0.0%)	
	**Median (IQR)**	10.74 (7.90-20.10)	12.85 (9.05-33.50)	
**Neutrophils x 10** ^9^ **/L**	**Neutrophilia**	17 (22.7%)	11 (45.8%)	0.044
	**Neutropenia**	3 (4.0%)	2 (8.3%)	
	**Median (IQR)**	4.60 (2.95-15.40)	6.5(4.70-21.00)	
**CRP (mg/dl)**	**Median (IQR)**	6 (2.40-9.90)	10.90 (2.15-8.60)	0.039
**Cause of pneumonia (** ***n*** **=99)**	**Viral pneumonia (** ***n*** **=35)**	33^a^ (44.0%)	2^b^ (8.3%)	<0.001
	**Bacterial pneumonia (** ***n*** **=20)**	10 ^a^(13.3%)	10^b^(41.7%)	
	**Viral bacterial co-infection (** ***n*** **=30)**	21 ^a^ (28.0%)	9 a (37.5%)	
	**Viral coinfection (** ***n*** **=11)**	10^a^(13.3%)	1^a^ (4.2%)	
	**Bacterial co-infection (** ***n*** **=3)**	1a (1.3%)	2^a^ (8.3 %)	
**Type of bacterial pathogens (** ***n*** **=64)**	**Gram-positive (** ***n*** **=7)**	6 (18.8%)	1 (4.8%)	0.024
	**Gram-negative (** ***n*** **=39)**	20 (62.5%)	19 (90.5%)	
	**Atypical bacteria (** ***n*** **=2)**	2 (6.3%)	0 (0.0%)	
	**Combined gram-positive and negative (** ***n*** **=5)**	4 (12.5%)	1 (4.8%)	
**Common viral pathogens (** ***n*** **=62)**	***RSV (n=28)***	26 (34.6%)	2 (8.3%)	0.008
	***Rhino virus (n=14)***	12 (15.2%)	2 (8.3%)	
	***Adenovirus (n=11)***	10 (14.6%)	1 (4.2%)	
	***HMPV (n=9)***	8 (10.1%)	1 (4.2%)	
**Common bacterial pathogens (** ***n*** **=57)**	***Streptococcus pneumoniae (n=11)***	9 (11.4%)	2 (8.3%)	1.000
	***Klebsiella pneumoniae (n=36)***	18 (22.8%)	18 (75.0%)	<0.001
	***Pseudomonas aeruginosa (n=5)***	1 (1.3%)	4 (16.7%)	0.010
	***Hemophilus influenzae (n=5)***	4 (5.1%)	1 (4.2%)	1.000
**Pattern of antibiotic resistance (** ***n*** **=36) **	**Susceptible (** ***n*** **=6)**	4 ^a^ (18.2%)	2 a (14.3%)	0.005
	**MDR (** ***n*** **=20)**	16 ^a^ (72.7%)	4 ^b^ (28.6%)	
	**XDR (** ***n*** **=9)**	2 ^a^ (9.1%)	7 ^b^ (50.0%)	
	**Susceptible& MDR (** ***n*** **=1)**	0 a (0.0%)	1 a (7.1%)	
**Need oxygen**	** Yes **	45 (60.8%)	24 (100.0%)	<0.001
**Need of MV **	**Yes **	9 (12.0%)	21(87.5%)	<0.001

*CHD *Congenital heart diseases, *IQR *Interquartile rang, *MV *Mechanical ventilation, *WBC *White blood cell count, *CRP *C-reactive protein, *MDR *Multi-drug resistant, *XDR *Extensive -drug resistant *Significant results≤.05

When comparing the laboratory parameters of deceased and survived children, the median CRP level [10.90 (2.15–8.60) mg/dl] and neutrophil [6.5 (4.70–21.00) × 10^9^/L] were both significantly higher in deceased children (*p* = 0.044 and 0.039, respectively). Additionally, 87.5% of deceased children required ventilator support, and all deceased children required oxygen supplementation upon admission (*p* < 0.015). Of survived pneumonia cases, 44.0% were caused by a single virus, whereas 41.7% of deceased cases were caused by bacteria (*p* < 0.001). *Klebsiella pneumoniae* was isolated in 75.0% of deceased cases, which was statistically significant (*p* < 0.001). Regarding the susceptibility pattern of isolated bacteria, infection with XDR was significantly higher (*p* = 0.005) among deceased children, while infection with MDR and susceptible bacteria was more common in survivors (72.7% and 18.2%, respectively) (Table 5). To study the independent factors of mortality, bivariate and multivariate logistic regression analysis was employed. Mortality was independently associated with the following risk factors: the need for mechanical ventilation (95%, CI: 12.711–207.308, *p* < 0.001), the presence of complications (95%, CI: 7.45–162.723, *p* = 0.026), and pulmonary hypertension (95%, CI: 1.045–14.907, *p* = 0.027) (Table 6).

**Table 6:** Bivariate and multivariable logistic regression analysis to assess independent risk factors of mortality among children with CHD and pneumonia

	**Unadjusted**				**Adjusted**			
	**Sig.**	**OR**	**95% CI**		**Sig.**	**OR**	**95% CI**	
**Comorbidities (ref=No)**	0.050*	2.571	.999	6.620	NS			
**Malnutrition (ref=No)**	<0.001*	7.158	2.575	19.900	NS			
**Pulmonary Hypertension(ref=No)**	0.043*	3.947	1.045	14.907	.027*	10.331	1.308	81.602
**Complications (ref=No) **	<0.001*	34.833	7.457	162.723	.026*	10.045	1.320	76.437
**Heart Failure(ref=No)**	<0.001*	8.714	3.116	24.371	NS			
**Need MV (ref=No)**	<0.001*	51.333	12.711	207.308	<.001*	55.283	7.546	405.009
**Pattern of resistance (ref=Susceptible) **	0.039*				NS			
**MDR**	0.640	0.625	0.087	4.491				
**XDR**	0.045*	7.000	1.693	70.743				
**Bacterial Cause of Pneumonia (ref= viral infection)**	0.018*	7.071	1.390	35.981	NS			

*NS *Not significant; **Significant results≤.05; *OR* Odds ratio. *MDR *Multi-drug resistant, *XDR *Extensive -drug resistant, *MV *Mechanical ventilation

Variable(s) entered on model’s first step: co-morbidities, complications, Malnutrition, Need of mechanical ventilation, pulmonary hypertension, Heart failure, causes of pneumonia, and resistance pattern; Model X2 52.180, p<.001*; R2 70.9%; Hosmer and Lemeshow Test, X21.263, p.868

## Discussion

To the best of our knowledge, this was the first study conducted at our institution that examined pneumonia in children with CHD. This study focused on common causes and outcome of children with CHD admitted due to pneumonia. Children with a-cyanotic heart diseases were the most prevalent (81.8%). Lower prevalence of CHD was detected in previous studies [17–19]. The current study’s high prevalence can be linked to its main emphasis on pneumonia in CHD. Among the study participants, the leading cause of pneumonia was Viral representing 46.5% of cases, followed by viral-bacterial co-infection at 30.3%, while bacterial infection was the least common at 23.2%. Healthcare professionals should maintain a strong suspicion for viral infections, particularly during peak seasons for respiratory viruses, and should consider prompt diagnostic testing and antiviral treatment when appropriate.

Different results were detected by El-Nawawy A et al., studied pneumonia in children admitted to the PICU, with 20% having CHD. They reported viral pneumonia in 28.5% of cases, while 63% had mixed infections ([20]). Similarly, Yang A, et al. ([21]) found that mixed infection (42%) followed by bacterial (26%) and viral infection (19%) were the most common causes of pneumonia. Consistent with the current research, a high prevalence of viral pneumonia was detected in Egypt ([22]) and in Senegal [23], while Nathan AM et al. ([24]) found that bacteria pathogens were the most common cause of pneumonia.

Differences in the studied groups; demographics, the severity of the pneumonia, and seasonal variability may all contribute to the variation in the cause of pneumonia between studies. Moreover, the microbiological methods used for identification of the cause of pneumonia can have impact. The current study focused mainly on children with pneumonia and CHD admitted to pediatric wards, while the other two studies included children admitted in the PICU with various comorbidities and pneumonia.

Differentiating bacterial pneumonia from viral pneumonia based on the clinical presentations is challenging as the clinical signs and symptoms overlap. In the present study, cough and wheezing were more common in viral pneumonia (*p* = 0.001 and 0.028, respectively). In contrast to tachypnea, cyanosis and the need for ventilator support were significantly prevalent in bacterial pneumonia (*p* = 0.002, 0.010, and < 0.001, respectively). Consistent findings were observed in other studies [25, 26], indicating that rhinorrhea and wheezing were more common in viral pneumonia; however, unlike the present study tachypnea was found to be more frequent in viral pneumonia cases. Nathan AM et al. [24] found that only crepitations were associated with bacterial infection. Differences in clinical presentation across various studies can be explained by multiple factors. First, age plays a significant role; infants under one year often present with non-specific symptoms like poor feeding. Second, disease severity can influence signs; in severe cases, especially among infants, respiratory fatigue may paradoxically result in subtle symptoms such as diminished tachypnea or a weak cough, despite serious lung involvement. Third, existing comorbidities like CHD can weaken respiratory function and reduce the effectiveness of the cough reflex. Lastly, the causative agents of pneumonia can also impact the clinical presentation.

*Respiratory syncytial virus* (31%), *Rhinoviru*s (15.6%), and *Adenovirus* (12%) were the most prevalent viruses. Similar findings were found by El-Nawawy et al. [20] where the most prevalent viruses were *RSV* and *Rhinovirus* (32.1% and 29.5%, respectively). Also, another multicenter study conducted in Egypt detected that *RSV* 63.8%, *Rhinovirus* 10%, *Influenza* 9%, and *Adenovirus* 5% were the most common viruses causing lower respiratory tract infections in children [22]. Different results were detected in Senegal where *Adenovirus* (50%), *Influenza* virus (45.7%,), *Rhinovirus* (40%), *Enterovirus* (25.3%) and *RSV* (16%) were the most common [23]. Seasonal variations, severity of pneumonia, and characteristics of the children included in every study may all have an impact on the viruses that cause pneumonia. Worldwide *RSV* is an important cause of lower respiratory tract infections among children and those with CHD are more vulnerable to severe infection [27]. In many countries, children with CHD receive *RSV* prophylaxis (e.g., palivizumab) as part of national guidelines [28, 29]. In Egypt, there are no widely established or implemented national guidelines for *RSV* prophylaxis in high-risk groups including children with CHD. Additionally, high cost with limited government or institutional funding makes access difficult for families.

Enhancing diagnostic tests for determining the cause of pneumonia is crucial for directing therapy and reducing mortality rates, particularly in children with CHD. In this study, multiplex PCR demonstrated higher sensitivity in detecting bacterial pathogens compared to traditional culture methods, with detection rate of 87.5% versus 64%. The multiplex PCR was the primary method in detecting *Streptococcus pneumoniae* (100%), *Haemophilus influenzae* (100%) and atypical bacteria (100%). The distinction between the two methods can be explained by the fact that PCR can detect both viable and non-viable bacteria, whilst culture method only identifies viable bacteria. PCR can also detect small amounts of genetic material. As a result, using PCR can improve the diagnosis of bacterial pneumonia, particularly following antibiotic therapy.

Gram-negative bacteria (78%) were the most common cause in CAP, with *Klebsiella pneumoniae* (56.3%), *Haemophilus influenzae*, and *Pseudomonas aeruginosa* (9.4% each) being the most prevalent. *Streptococcus pneumoniae* (17.2%) was the most prevalent gram-positive bacteria, while atypical bacteria were the least common. Gram-negative bacteria, including *Klebsiella pneumoniae* (71.4%), *Pseudomonas aeruginosa (*21.4%) and *Acinetobacter* (7%) were the major cause of HAP. Similarly, Nathan AM et al. in Malaysia [24], who studied severe CAP pneumonia, concluded that *Haemophilus influenzae* (29.3%), *Staphylococcus aureus* (24%) and *Streptococcus pneumoniae* (22.7%) were the most common. Similar findings were detected in Egypt [30], found that *Klebsiella pneumoniae* (79.4%), *E. coli* (42.4%) and *Staph hominis (39.7%)* were the common bacteria causing CAP in children with CHD. Also, *Klebsiella pneumoniae* (81.8%), *E. coli*, *Staph hominis* and *Acinetobacter* (9% each) were the leading causes of HAP. In another study in Egypt [4], the cause of pneumonia in CHD was identified in 38.2% using blood culture with *Klebsiella pneumoniae* (47.6%) and *coagulase-negative Staphylococcus* aureus (23.8%) were the most prevalent. Moreover, *Streptococcus pneumoniae* (33%) and *Klebsiella pneumoniae* (20%) were the most common cause of CAP among children with comorbidity in China [21]. Assane D et al. [23] detected that *Streptococcus pneumoniae* (17.9%), *M. catarrhalis* (15.43%), and *H. influenzae* (8%) were the most common.

The disparity in the etiologies of pneumonia between different studies could be attributed to demographic characteristics of studied cases, seasonal, geographical difference, different microbiological methods used for identification of the cause of pneumonia, the difference in samples used for diagnosis. The current study’s high prevalence of gram-negative bacteria could be explained by the fact that 11% of pneumonia cases were HAP, and the current study focused only on children with CHD. Furthermore, lack of routine immunization against *Streptococcus pneumonia* in Egypt is a significant contributor to its frequency, particularly in children with CHD.

Children with viral- bacterial co-infections and bacterial infections had an increased risk of heart failure (*p* = 0.032), higher need for mechanical ventilation (*p* ≤ 0.001), longer hospital stay (*p* = 0.003) and a higher risk of mortality (*p* < 0.001) than those with viral pneumonia. Comparable results were observed in other studies [31–33]. CHD has deleterious effects on the lungs, including altered lower airway resistance, disrupted surfactant composition, and lung injury caused by excessive or insufficient blood flow. Furthermore, respiratory infections in children with cardiac abnormalities might result in heart failure and/or pulmonary hypertensive crisis. Finally, reduced cardiac function capacity limits the ability to increase cardiac output and oxygen delivery, which is exacerbated by compromised oxygen uptake because of increased respiratory effort and airway inflammation, all of which can be contributing factors in the current study, which focused primarily on CHD [18, 19]. 

A local antibiotic policy should be established based on the susceptibility patterns of isolated bacteria at both institutional and national levels to aid in the prompt and proper treatment of pneumonia in children with CHD. In the present study, there was an alarming increase in the resistant pattern of isolated bacteria. In CAP 72.4% of bacterial isolates were MDR and 66.7% of bacterial isolates causing HAP were XD. *Klebsiella pneumoniae* showed the highest resistance rates in both CAP and HAP. The emergence of carbapenem-resistant *pseudomonas aeruginosa* has been an increasing problem in many parts of the world [34, 35]. Our results showed an alarming increase in carbapenem resistance among *pseudomonas aeruginosa* in CAP; 50% of isolates were resistant to meropenem. While all isolates were sensitive to cefepime, piperacillin/tazobactam, amikacin and quinolones. Previous studies in Egypt also detected high resistance rates in bacteria causing pneumonia among children with CHD [4, 30]. In contrast, study in China [36], *Klebsiella pneumoniae* showed lower resistance rate while *Pseudomonas aeruginosa* showed higher resistance. Also, lower resistance of *Klebsiella Pneumoniae and pseudomonas aeruginosa* was detected in Korean surveillance of CAP in children and adolescents [37]. 

All *Streptococcus pneumoniae* isolates were resistant to penicillin, macrolides and trimethoprim sulfamethoxazole, and half of isolates were resistant to 3rd generation cephalosporine. Furthermore, approximately one-third of isolates were resistant to quinolones and clindamycin. High resistance to macrolides (93.5%) was detected also in Korea and 3rd generation cephalosporin (8.7%) and penicillin (14%) showed lower resistance compared to current study [37]. 

The high resistance rate in the current study could be explained by the fact that children with CHD are more likely to be colonized with resistant pathogens as they frequently visit the health care facilities for their heart problems and recurring infections that are treated with multiple courses of antibiotics. Additionally, the current analysis included HAP in 11% of cases, and 66.7% of isolates causing HAP were XDR.

This alarming increase in resistant rate emphasizes the importance of establishing antimicrobial stewardship to help in optimizing antibiotic prescription. These results will help in establishing an institutional antibiogram to help in guiding empiric antibiotics therapy in children with CHD presented with pneumonia, which could reduce both morbidity and mortality. According to the antimicrobial susceptibility data represented in the current study, carbapenem (especially meropenem) in combination with quinolones could be used as empiric therapy for treatment of CAP in children with CHD until culture results are available but further multicenter studies are needed to help in establishment of local guidelines.

In the present study, 23.3% of cases decreased. High mortality among children with CHD admitted with pneumonia was also detected in other studies [20, 38, 39]. The high mortality rate could be attributed to the study’s concentration on children with CHD hospitalized due to pneumonia. Additionally, our institute serves as a tertiary care referral center that admits severe and complicated cases and 30% of studied cases required ventilator support indicating the severity of pneumonia. This highlights the importance of implementing pneumonia preventive measures among children with CHD.

Tachypnea (*p* = 0.025), difficulty in breathing (*p* = 0.019) and cyanosis(*p* < 0.001) were common among deceased children. Furthermore, median WBC count (*p* = 0.044), median neutrophil count (*p* = 0.044) and median CRP (p = o, o39) were higher among deceased children. Similar findings were concluded by Shan et al. [38] studying the risk factors of mortality among children with pneumonia; abnormal white blood cells, and C-reactive protein results were independent risk factors for both intensive care unit admission (ICU) admission and poor clinical. Children presented with symptoms of respiratory distress on admission are at an increased risk of ICU admission. Mortality among the cases studied was independently related to the presence of pulmonary hypertension that increased mortality by four times (95% CI: 1.045–14.907, *p* = 0.027), also complications increased the odds of mortality 35- times (95% CI: 7.45–162.723.45.723, *P* = 0.026) and the need for mechanical ventilation increased the odds of mortality 51 times (95% CI: 12.711–207.308.711.308, *P* < 0.001). In a systemic analysis of 143 studies [39], hypoxemia, decreased conscious state, severe acute malnutrition, and the presence of an underlying chronic illness as CHD were all consistently related to increased mortality in children with pneumonia.

The current study was not without limitations; this single-center observational study leading to relatively small sample size leading to limitations in studying the risk factors of mortality. Detection of microorganisms using PCR suggested presumed bacterial or viral pneumonia, and may not confirm causality, particularly considering the limitations of the sample type.

## Conclusions

The present study provides a thorough evaluation of pneumonia in pediatric patients with CHD, revealing prevalent causes and outcomes. Bacterial confection can co-exist even when a viral pathogen is isolated. Gram-negative bacteria, especially *Klebsiella pneumoniae*, are a major cause of pneumonia in children with CHD. The combination of culture techniques and multiplex PCR was beneficial in the accurate and timely identification of the causative agents of pneumonia in this vulnerable population that helps in guiding therapy. Pulmonary hypertension, comorbidities, and the need of mechanical ventilation are independent risk factors for death among children with CHD admitted with pneumonia. Enhancing *Streptococcus pneumoniae* vaccination among children with CHD and the use of *RSV* prophylaxis could help in decreasing pneumonia episodes.

## Data Availability

Most data analyzed during this study are included in this published article. The underlying datasets used and/or analyzed during the current study are available from the corresponding author on reasonable request.

## Abbreviations

CRP: C-reactive protein
CAP: Community-acquired pneumonia
HAP: Hospital-acquired pneumonia
HMPV: Human Metapneumovirus
MDR: Multi-drug resistant
PCR: Polymerase Chain Reaction
PDR: Pan-drug resistant
PICU: Pediatric Intensive Care Unit
RSV: Respiratory Syncytial Virus
XDR: Extensively drug-resistant
